# A single-center, retrospective study of hospitalized patients with lower respiratory tract infections: clinical assessment of metagenomic next-generation sequencing and identification of risk factors in patients

**DOI:** 10.1186/s12931-024-02887-y

**Published:** 2024-06-20

**Authors:** Qinghua Gao, Lingyi Li, Ting Su, Jie Liu, Liping Chen, Yongning Yi, Yun Huan, Jian He, Chao Song

**Affiliations:** 1 Department of Pulmonary and Critical Care Medicine, Anning First People’s Hospital Affiliated to Kunming University of Science and Technology, Kunming, 650302 China; 2 Department of Medical, Hangzhou Matridx Biotechnology, Hangzhou, 311100 China; 3 Department of Medical Imaging, Anning First People’s Hospital Affiliated to Kunming University of Science and Technology, Kunming, 650302 China

**Keywords:** Metagenomic next-generation sequencing (mNGS), Lower respiratory tract infections (LRTIs), Diagnostic efficacy, Predictive model, Nomogram

## Abstract

**Introduction:**

Lower respiratory tract infections(LRTIs) in adults are complicated by diverse pathogens that challenge traditional detection methods, which are often slow and insensitive. Metagenomic next-generation sequencing (mNGS) offers a comprehensive, high-throughput, and unbiased approach to pathogen identification. This retrospective study evaluates the diagnostic efficacy of mNGS compared to conventional microbiological testing (CMT) in LRTIs, aiming to enhance detection accuracy and enable early clinical prediction.

**Methods:**

In our retrospective single-center analysis, 451 patients with suspected LRTIs underwent mNGS testing from July 2020 to July 2023. We assessed the pathogen spectrum and compared the diagnostic efficacy of mNGS to CMT, with clinical comprehensive diagnosis serving as the reference standard. The study analyzed mNGS performance in lung tissue biopsies and bronchoalveolar lavage fluid (BALF) from cases suspected of lung infection. Patients were stratified into two groups based on clinical outcomes (improvement or mortality), and we compared clinical data and conventional laboratory indices between groups. A predictive model and nomogram for the prognosis of LRTIs were constructed using univariate followed by multivariate logistic regression, with model predictive accuracy evaluated by the area under the ROC curve (AUC).

**Results:**

**(1) Comparative Analysis of mNGS versus CMT**: In a comprehensive analysis of 510 specimens, where 59 cases were concurrently collected from lung tissue biopsies and BALF, the study highlights the diagnostic superiority of mNGS over CMT. Specifically, mNGS demonstrated significantly higher sensitivity and specificity in BALF samples (82.86% vs. 44.42% and 52.00% vs. 21.05%, respectively, *p* < 0.001) alongside greater positive and negative predictive values (96.71% vs. 79.55% and 15.12% vs. 5.19%, respectively, *p* < 0.01). Additionally, when comparing simultaneous testing of lung tissue biopsies and BALF, mNGS showed enhanced sensitivity in BALF (84.21% vs. 57.41%), whereas lung tissues offered higher specificity (80.00% vs. 50.00%). **(2) Analysis of Infectious Species in Patients from This Study**: The study also notes a concerning incidence of lung abscesses and identifies *Epstein-Barr virus* (EBV), *Fusobacterium nucleatum*, *Mycoplasma pneumoniae*, *Chlamydia psittaci*, and *Haemophilus influenzae* as the most common pathogens, with Klebsiella pneumoniae emerging as the predominant bacterial culprit. Among herpes viruses, EBV and herpes virus 7 (HHV-7) were most frequently detected, with HHV-7 more prevalent in immunocompromised individuals. **(3) Risk Factors for Adverse Prognosis and a Mortality Risk Prediction Model in Patients with LRTIs**: We identified key risk factors for poor prognosis in lower respiratory tract infection patients, with significant findings including delayed time to mNGS testing, low lymphocyte percentage, presence of chronic lung disease, multiple comorbidities, false-negative CMT results, and positive herpesvirus affecting patient outcomes. We also developed a nomogram model with good consistency and high accuracy (AUC of 0.825) for predicting mortality risk in these patients, offering a valuable clinical tool for assessing prognosis.

**Conclusion:**

The study underscores mNGS as a superior tool for lower respiratory tract infection diagnosis, exhibiting higher sensitivity and specificity than traditional methods.

## Introduction

Lower respiratory tract infections (LRTIs) are a major global contributor to morbidity and mortality [1]. A wide variety of pathogens, including bacteria, viruses, fungi, mycoplasma, and parasites, contribute to the occurrence of LRTIs [2]. The prompt and accurate identification of pathogens facilitates the implementation of targeted treatments, minimizes the utilization of broad-spectrum antibiotics, and mitigates the disease burden on patients [3, 4]. While traditional pathogen cultivation methods have long been regarded as the gold standard for microbial identification, their time-consuming nature and limited sensitivity, particularly when dealing with challenging-to-culture bacteria, viruses, atypical pathogens, and rare pathogens, often result in diagnostic and treatment delays [5].

Metagenomics Next Generation Sequencing (mNGS), also referred to as high-throughput sequencing, has a sequencing depth ranging from 10 to 20 million sequences per sample. MNGS can detect a wide range of clinical samples, including peripheral blood, cerebrospinal fluid, throat swabs, sputum, bronchoalveolar lavage fluid (BALF), tissues, urine, and feces [6, 7]. BALF serves as a suitable specimen for pathogen detection in LRTIs. Lung biopsy is commonly employed for distinguishing between lung cancer and tuberculosis. In cases where BALF is insufficient for diagnosing *Mycobacterium tuberculosis complex* (MTBC), lung tissue can serve as a viable alternative [8]. In the diagnosis of localized infections, noninvasive approaches are less sensitive than biopsy and cannot distinguish between colonizing commensals and invasive pathogens [9]. Additionally, lung biopsy provides a more accurate assessment of immune competence than noninvasive methods and is necessary to rule out infection and diagnose noninfective conditions, such as chemotherapy-induced acute lung injury [10]. However, the performance of mNGS can vary depending on sample types, sample sizes, and populations, and the accuracy of sampling may impact the results of mNGS. Several retrospective studies on mNGS-based detection of LRTIs have demonstrated its superior detection performance compared to traditional pathogen monitoring methods [11–15]. The detection of herpesviruses is exceedingly common in mNGS reports. Notably, species from the herpesvirus family, including *Epstein-Barr virus* (EBV), *cytomegalovirus* (CMV), and herpes simplex virus, are the most commonly identified viruses in these studies [16, 17]. Herpesviruses can induce pulmonary diseases in respiratory tract infections, particularly in immunocompromised individuals. Furthermore, they have been linked to the development and advancement of bronchiectasis, potentially via alternative biological pathways like telomere depletion and cell senescence [16]. Herpes viruses can induce pulmonary diseases in respiratory tract infections, particularly in immunocompromised individuals. Furthermore, they have been linked to the development and advancement of bronchiectasis, potentially via alternative biological pathways like telomere depletion and cell senescenc. MNGS enhances the detection rate of herpes viruses; however, the characteristics of distinct herpes virus subtypes and their associations with diseases, as well as the underlying mechanisms, remain unclear. MNGS exhibits characteristics such as comprehensiveness, high throughput, high sensitivity, and unbiasedness, making it extensively utilized in the field of clinical pathogen microbiology detection [18, 19]. Therefore, the evaluation and optimization of this technology’s performance are of utmost importance.

This retrospective study was conducted at the Department of Respiratory and Critical Care Medicine of the First People’s Hospital of Anning City between July 2020 and July 2023, which included 451 patients with suspected LRTI. It aimed to compare the specificity and sensitivity of mNGS with conventional microbiological testing (CMT), to analyze the distribution of pathogen profiles in patients with LRTI, to compare the detection performance of lung tissue biopsies and BALF samples in mNGS, and to count the frequency of herpesvirus family members. The study also developed a prediction model based on lymphocyte percentage, underlying disease, traditional laboratory test results, and herpesvirus test results to provide more accurate information and guidance for the diagnosis, prognosis, and treatment of LRTIs, and herpesvirus test results to provide more accurate information and guidance for the diagnosis, prognosis, and treatment of LRTIs.

## Methods

### Patients and study design

This retrospective cohort study encompasses patients suspected of LRTIs who underwent bronchoscopy and mNGS at the Department of Pulmonary and Critical Care Medicine, Anning First People’s Hospital Affiliated to Kunming University of Science and Technology, between July 2020 and July 2023. LRTIs were defined in accordance with the Guidelines for the Management of Adult Lower Respiratory Tract Infections [20] as acute conditions lasting 21 days or less, predominantly featuring cough, accompanied by at least one other symptom of the lower respiratory tract—such as expectoration, dyspnoea, wheeze, or chest pain/discomfort—without alternative diagnoses like sinusitis or asthma. The study underwent ethical review by the Ethics Committee of the Anning First People’s Hospital Affiliated to Kunming University of Science and Technology (2022-025-01). We affirm that this study complies with the ethical principles outlined in the Helsinki Declaration, and all patient-related data are treated with strict confidentiality. As this study is retrospective and the data are analyzed anonymously, informed consent was not necessary.

Exclusion criteria were: (1) Age less than 14 years old; (2) No follow-up within 28 days after admission. The collected data comprised age, gender, underlying diseases, outcomes, time from admission to mNGS testing window, and laboratory results including white blood cell count, C-reactive protein (CRP) level, procalcitonin (PCT) level, neutrophil percentage (%), lymphocyte percentage (%), and CD4^+^ T cell count. The overall mortality rate was selected as the prognostic indicator in this study. Underlying diseases were categorized as pulmonary diseases [including chronic obstructive pulmonary disease (COPD), bronchiectasis, pulmonary fibrosis, pulmonary embolism, pulmonary hypertension, and post-tracheotomy); cerebrovascular diseases (including congestive heart failure, rheumatic heart disease, and congenital heart disease (atrial septal defect, ventricular septal defect)]; patients at risk of aspiration (including Alzheimer’s disease, cerebral infarction, cerebral hemorrhage, coma, epilepsy, prolonged bed rest, hiatal hernia, and reflux esophagitis); metabolic diseases (including diabetes mellitus); and immunodeficiency (including primary immunodeficiency, viral infections (*Influenza A virus*, SARS-CoV-2), malnutrition, hypoalbuminemia, and rheumatic autoimmune diseases). Malignant tumors referred to patients diagnosed with cancer before admission. Patients with two or more of the mentioned underlying diseases were classified as having mixed multiple underlying diseases. Patients without any underlying diseases were categorized as none. Other pathogen detection methods, except for routine microbial culture and smear, were only conducted on patients highly suspected of relevant infections. The respiratory six-panel reagent kit was used for fluorescent quantitative polymerase chain reaction(PCR) detection of SARS-CoV-2, *Influenza A virus, Influenza B virus, Influenza C virus*, *Human Adenovirus*, *Respiratory syncytial virus*(RSV), *Human metapneumovirus*(HMPV). Galactomannan(GM) and (1–3)-β-d-glucan test were conducted for suspected fungal infections, and GeneXpert was performed for patients suspected of tuberculosis. The final diagnosis was established by a consensus of three experienced clinicians, who integrated all microbiological test results and the patient’s response to treatment.

### specimen collection and processing

Initially, patients’ tolerance for bronchoscopy and lung biopsy was confirmed. Subsequently, when clinicians suspected complex infections or potential tumors, rapid on-site evaluation (ROSE) was conducted, but differentiation among fungal, tuberculosis, anaerobic bacterial, or specific pathogen infections proved challenging. Given the limitations of relying solely on attending physicians’ experience and BALF for accurate etiological identification, both BALF and tissue samples were concurrently collected for mNGS. The specimens were stored at − 20 °C for mNGS.

### MNGS testing

1) Pretreatment and Nucleic Acid Extraction.

To each 3 × 3 × 3mm^3^ tissue sample, PBS was added to achieve a final volume of 1.2 mL. The samples were then manually ground and subjected to ultrasonic disruption for 5 min. Subsequently, 1.2 mL of the vortexed BALF was transferred to a 2 mL shaking tube containing glass beads of varying sizes for further disruption by vibration at a frequency of 30 Hz for 10 min. Both types of samples were then centrifuged at 12,000 rpm for 3 minutes. Afterward, 1 mL of the vortexed liquid was aspirated and used for nucleic acid extraction using a magnetic bead-based nucleic acid extraction kit (Matridx catalog number MD014).

2) Library Preparation and Sequencing.

DNA or RNA sequencing was performed on BALF samples. For RNA library, reverse transcription was conducted, followed by enzymatic fragmentation (excluding plasma), end repair, adapter and index ligation to prepare PCR-free libraries. Library concentration was determined using real-time PCR (KAPA) and pooled. Shotgun sequencing was performed on the Illumina NextSeq platform. Each library generates approximately 20 million 50 bp single-end reads.

3) Analysis of Raw Sequencing Data Using Bioinformatics Pipeline.

The following steps were primarily involved in the analysis of raw sequencing data using a bioinformatics pipeline:

(1) Removal of unnecessary adapter sequences and low-quality bases (Q-score cut, 20) in the pipeline.

(2) Mapping of human host sequences to the human reference genome (GRCh38.p13, https://www.ncbi.nlm.nih.gov/assembly/2334371) using BWA (Burrows-Wheeler alignment) to exclude human host sequences.

(3) After filtering out low-complexity reads, the remaining sequencing data is aligned against reference databases (NCBI nt database and GenBank [21]) using BWA to determine microbial species.

4) mNGS Reporting Standards.

Microbial reads identified from the library were reported if the following conditions were met:

(1) Sequencing data passes quality control filtering (library concentration > 50 pM, Q20 > 85%, Q30 > 80%).

(2) The negative control (NC) in the same sequencing run did not contain the species or RPM (sample)/RPM (NC) > 5 [22–24]. This value was empirically determined as a threshold to distinguish true positives from background contamination.

### Data analysis

Continuous variables were presented as mean ± standard deviation (SD). Categorical variables were indicated as the number of patients and their proportions.We evaluated the diagnostic performance of mNGS and CMT for the final clinical diagnosis (reference standard) by assessing sensitivity, specificity, positive predictive value (PPV), negative predictive value (NPV), and total consistency rate (TCR). We constructed a matrix based on the true positive, true negative, false positive, and false negative results of mNGS and CMT. The chi-square test and *p*-value with degrees of freedom were calculated using the chi2_contingency function in Python’s scipy package. Numerical variables did not follow a normal distribution. We employed the Wilcoxon rank-sum test for continuous variables and the Chi-square test for categorical variables. We conducted all statistical analyses using R software (v3.6), considering *p* < 0.05 (two-tailed) as significant. The regression analysis consisted of logistic regression, initially univariate analysis, followed by multivariable modeling. As the sample size did not exceed 500 cases, the variable selection principle for univariate followed by multivariable analysis was to include those with a univariate regression *P* < 0.10 to identify statistically significant risk factors influencing the recovery of patients with LRTIs and to select independent risk factors affecting the prognosis of these patients. The independent risk factors were utilized to develop a nomogram prediction model using the “rms” package in R software.

### Sensitivity analysis

Sensitive analysis was performed. This study reports the outcomes of patients after mNGS detection, including mortality or recovery.

## Results

### Patients and sample characteristics

**Fig. 1:**
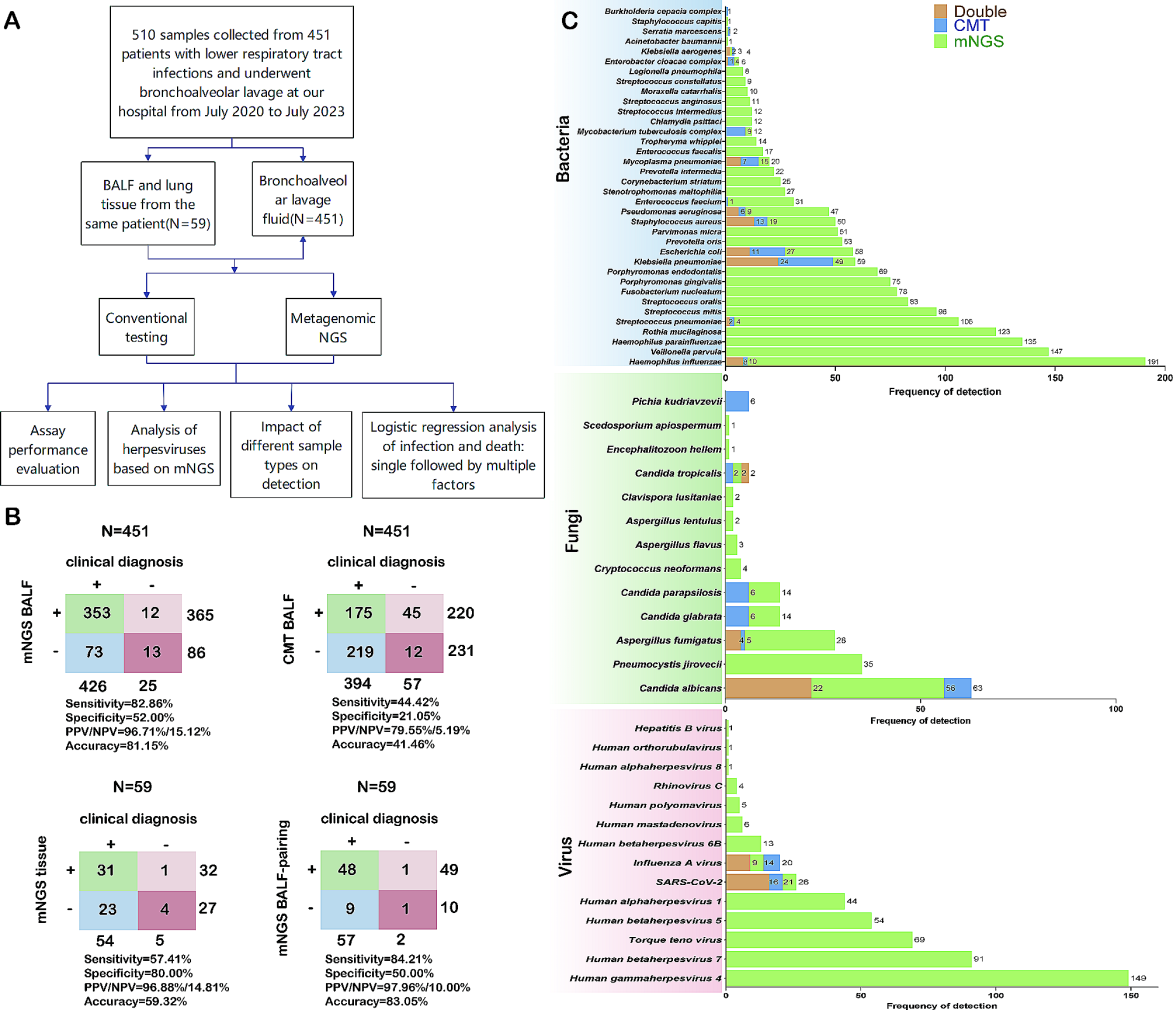
**A.** Contribution of mNGS testing to the diagnosis of patients with lower respiratory tract infections. A.Enrollment details and study design. **B.** Diagnostic performance of mNGS and CMT for pathogen detection (upper panel) and diagnostic performance of mNGS in paired lavage fluid and lung tissue samples (lower panel). **C.** Bar graph comparing pathogen detection between mNGS and culturing, bacterial spectrum comparison between mNGS and CMT, fungal spectrum comparison between mNGS and CMT, and comparison of viruses identified by mNGS and CMT

As shown in Fig. 1A, a retrospective study included 510 samples from 451 patients, consisting of 451 BALF samples. Among these, 59 patients underwent both BALF and lung biopsy tissue testing. The clinical and demographic characteristics of the patients are detailed in Table 1, with 320 male patients (71.00%) and 131 female patients (29.00%), and a median age of 63 years. The levels of CRP (median 36.05 mg/L), PCT (median 0.50 ng/mL), and neutrophil percentage (76.45%) were elevated compared to normal levels, lymphocyte percentage (11.35%) and CD4^+^ T cell count (242.27 cells/µL) were decreased, indicating overall elevated infection markers and reduced cellular immunity. Chronic lung diseases represented the highest proportion at 29.3% (132 cases). Eighty-two cases (18.2%) involved mixed underlying diseases, and the patients had a relatively older age and multiple underlying conditions, with 16 cases (3.50%) at risk of aspiration. Integrating all pathogen results, clinical pharmacotherapy responses, and the medical team’s diagnoses, 436 out of 451 patients suspected of LRTIs were ultimately diagnosed with infections, while 15 were finally diagnosed with non-infected diseases., including tumour (*n* = 7), organizing pneumonia (*n* = 3), COPD (*n* = 2), bronchial asthma (*n* = 1),pneumoconiosis (*n* = 1), bronchiectasis (*n* = 1).Among the infected patients, 69.80% had bacterial infections (*n* = 315), 5.10% had fungal infections (*n* = 23), 2.90% had viral infections (*n* = 13), and 18.80% had mixed infections (*n* = 85). In mixed infections, 11 patients were found to have concurrent viral and fungal infections, with 8 cases testing positive for *SARS-CoV-2*, 2 cases with *Aspergillus infection*, 4 cases with *Candida* infection, and 2 cases with *Pneumocystis jirovecii* infection. Additionally, 3 cases tested positive for influenza virus, with 2 cases also showing *Aspergillus fumigatus* infection and 1 case with *Pneumocystis jirovecii*. There were 14 cases of viral and bacterial co-infections, primarily involving SARS-CoV-2, influenza A virus, and *human parainfluenza virus*. One case involved bacterial and atypical pathogen co-infection, with Orientia tsutsugamushi and Streptococcus pneumoniae detected by mNGS. There were 47 cases of bacterial and fungal co-infections. A total of 12 patients had concurrent bacterial, fungal, and viral infections, with 10 cases positive for SARS-CoV-2 and 2 cases positive for influenza A. These cases predominantly involved Aspergillus fumigatus, Candida, and Pneumocystis jirovecii, accompanied by high abundances of common bacteria associated with community-acquired pneumonia (CAP) and hospital-acquired pneumonia (HAP). Almost all patients with these multiple infections had underlying diseases. In terms of outcomes, 94.20% of patients (*n* = 425) showed improvement after treatment and were discharged, while 5.80% (*n* = 26) died. According to the mNGS test results, 52.80% of patients were positive for herpes virus (*n* = 238), while 47.20% (*n* = 213) were negative.

**Table 1:** Demographic and clinical characteristics of patients included in this study interquartile range[IQR]

Variable	level	Overall
*n*		451
Age (median [IQR])		63.00 [46.00, 75.00]
CRP (median [IQR])	Range(< 10 mg/L)	36.05 [8.85, 93.33]
PCT (median [IQR])	Range(0–0.046 ng/mL)	0.50 [0.22, 0.81]
WBC 10^9/L(median [IQR])	Range(3.5–9.5×10^9/L)	7.76 [5.37, 11.51]
Ratio of neutrophils (median [IQR])	Range(40–75%)	76.45 [63.40, 86.20]
Ratio of lymphocytes(median [IQR])	Range(20–50%)	11.35 [5.60, 19.50]
CD4 (median [IQR])	Range(706–1125/µL)	242.27 [78.78, 463.30]
Days from admission to mNGS testing (median [IQR])		2.00 [1.00, 4.00]
Sex (%)	Female	131 (29.00)
	Male	320 (71.00)
Underlying.disease (%)	Cardiovascular and cerebrovascular diseases	27 (6.00)
	Chronic lung diseases	132 (29.30)
	Comorbidity with multiple underlying diseases	82 (18.20)
	Immunosuppressive	30 (6.70)
	Malignant tumour	28 (6.20)
	Metabolic diseases (diabetes)	48 (10.60)
	None	88 (19.50)
	Risk of aspiration	16 (3.50)
mNGS (%)	False negative	73 (16.20)
	False positive	12 (2.70)
	True negative	13 (2.90)
	True positive	353 (78.30)
CMT (%)	False negative	219 (48.60)
	False positive	45 (10.00)
	True negative	12 (2.70)
	True positive	175 (38.80)
Infection (%)	No	15 (3.30)
	Yes	436 (96.70)
Non-infected diseases(%)	Tumour	7(46.67)
	Organizing pneumonia	3(20.00)
	COPD	2(13.33)
	Bronchial asthma	1(6.67)
	Pneumoconiosis	1(6.67)
	Bronchiectasis	1(6.67)
Infection.types (%)	Bacterial	315 (69.8)
	Fungal	23 (5.1)
	Mixed infection	85 (18.8)
	Non-infectious disease	15(3.3)
	Viral	13 (2.9)
Outcome (%)	Improvement	425 (94.2)
	Mortality	26 (5.8)
Herpes.virus.positive (%)	No	213 (47.2)
	Yes	238 (52.8)

mNGS, metagenomic next-generation sequencing

SD, standard deviation

IQR, interquartile range

CMT, conventional method test

AECOPD, acute exacerbation of chronic obstructive pulmonary disease

The false positive and false negative rates of mNGS in infected patients were 2.29% (10/436) and 16.74% (73/436), respectively, with the predictive accuracy of mNGS lower than the 90% threshold reported in many articles. In non-infected patients, the true negative rate of mNGS was relatively high at 86.67%, but there were also two false positive cases, including detection of EBV and human *herpes virus 7*(HHV-7),and *Candida albicans* with co-infection of EBV, *herpes simplex virus 1*(HSV-1), and HHV-7. In this study, instances of false negative mNGS but true positive CMT results were primarily associated with infections involving *Klebsiella pneumoniae* (*n* = 3), *Escherichia coli* (*n* = 3), *Staphylococcus aureus* (*n* = 1), *Pseudomonas aeruginosa* (*n* = 1), *Acinetobacter baumannii* (*n* = 2), *Candida albicans* (*n* = 2), *Mycoplasma pneumoniae* (*n* = 1), *influenza A virus* (*n* = 1), and SARS-CoV-2 (*n* = 2). All clinical samples underwent testing using CMT, and the diagnostic performance of mNGS and CMT in LRTIs is illustrated in Fig. 1B. In BALF, mNGS demonstrated a sensitivity of 82.86% and a specificity of 52.00%, with positive predictive value and negative predictive value of 96.71% and 15.12%, respectively. In comparison, CMT showed a sensitivity of 44.42% and a specificity of 21.05%, with positive predictive value and negative predictive value of 79.55% and 5.19%, respectively. The overall specificity, sensitivity, positive predictive value, and negative predictive value of mNGS were significantly higher than those of traditional methods (*χ*^2^ = 152.15, *p* < 0.001). When examining paired samples, the sensitivity of mNGS in tissue and BALF was 57.41% vs. 84.21% and the specificity was 80.00% vs. 50.00%. The positive predictive values were 96.88% vs. 97.96%, and the negative predictive values were 14.81% vs. 10.00%. In general, the accuracy of lavage fluid was superior (83.05% vs. 59.32%) (*χ*^2^ = 11.58, *p* < 0.001).

### The distribution of pathogens among patients

Figure 1C illustrates the pathogen distribution among patients in our facility (test results, not diagnostic results). Among bacterial pathogens, *Klebsiella pneumoniae*, *Haemophilus influenzae, Streptococcus pneumoniae, Pseudomonas aeruginosa, Staphylococcus aureus*, and *Mycoplasma pneumoniae* were the most commonly detected, identified through a combination of CMT and mNGS. Among fungal pathogens, *Candida albicans, Aspergillus fumigatus*, and *Pneumocystis jirovecii* exhibited relatively higher detection rates. Notably, *Pichia kudriavzevii* was only detected through culture but not identified by mNGS. Regarding viral pathogens, *human herpesvirus 4, human herpesvirus 7, picornavirus, human herpesvirus 5, herpes simplex virus type 1*, SARS-CoV-2, and *influenza A virus* showed relatively higher detection rates. SARS-CoV-2 and *influenza A virus* are typically detected using qPCR or antigen testing in CMT. It is noteworthy that none of the cases with identified herpesviruses in this retrospective analysis received specific antiviral treatment for herpesviruses. MNGS detected a broader range of pathogens compared to CMT. While most pathogens identified by CMT were also identifiable by mNGS, there were instances where CMT produced true positives whereas mNGS generated false negatives. Instances of this discrepancy were predominantly noted in the isolation of *Klebsiella pneumoniae* and *Klebsiella oxytoca* from BALF, where mNGS failed to identify the specific species. Additionally, two cases showed positive IgM for *Mycoplasma pneumoniae* in serum, resulting in the clinical diagnosis of *Mycoplasma pneumonia*, despite no *Mycoplasma* species being detected by mNGS. Moreover, *Escherichia coli* was cultured from BALF in one patient, whereas mNGS identified *Escherichia coli* but did not report it. The discrepancy could be due to the frequent presence of *Escherichia coli* as a contaminant in mNGS reagents and its extensive prevalence in wet laboratory reagents. A relatively high reporting threshold for *Escherichia coli* in mNGS may lead to its filtration and subsequent false negatives.

### Differences between mNGS testing and BALF sampling

**Fig. 2:**
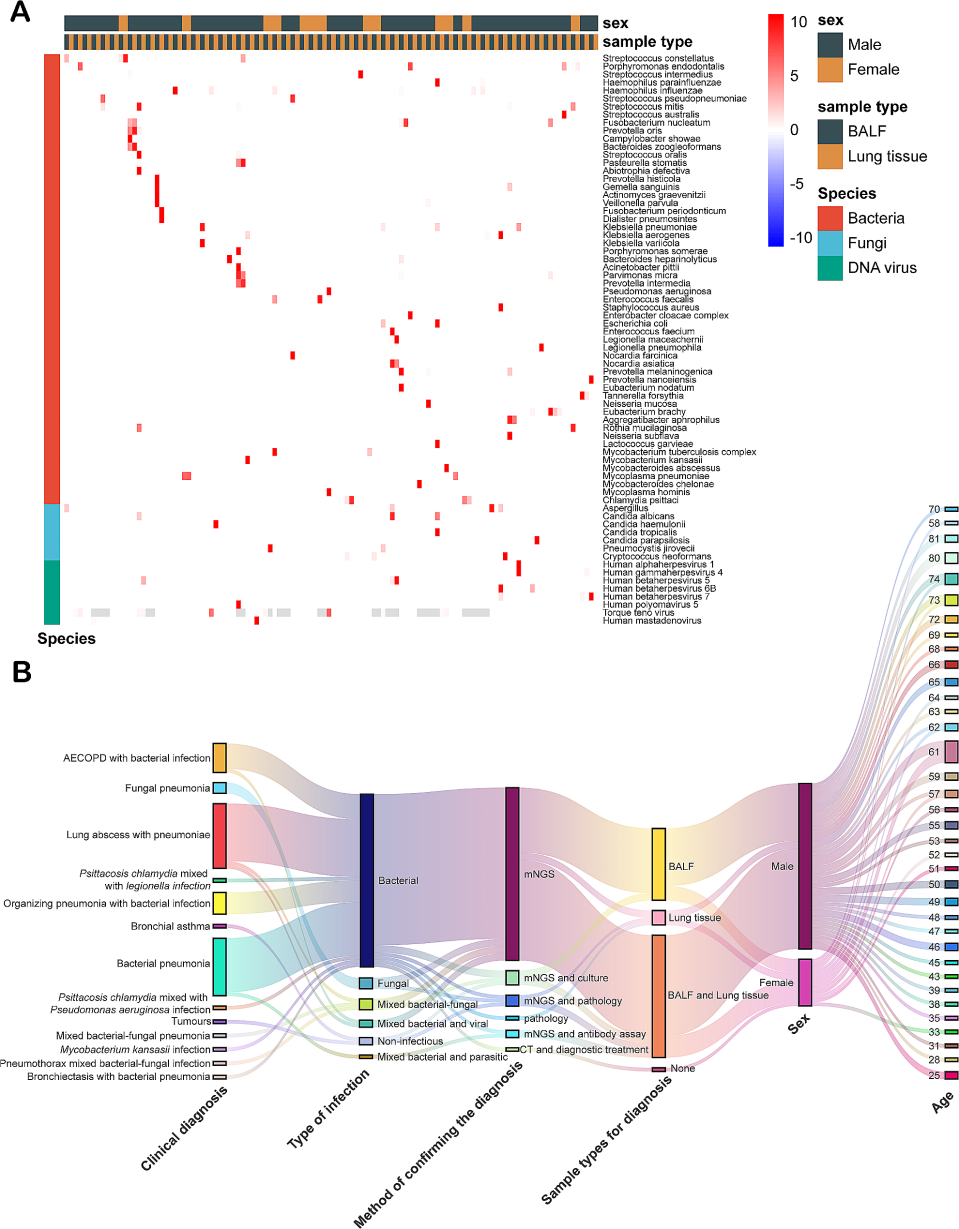
Results of mNGS testing on BALF and synchronous lung tissue samples. (**A**) Heatmap of species sequencing numbers detected in lavage fluid and tissue samples (sequencing numbers were normalized). (**B**) Feature Sankey diagram of tissue detection. Frequency and relative abundance of herpesvirus infections

Fifty-nine cases underwent pathogen distribution testing in both tissue and BALF samples (Fig. 2A). Identified bacteria comprised *Propionibacterium acnes*, *Staphylococcus aureus*, *Streptococcus pneumoniae*, *Lactococcus lactis*, and yeast, typically normal flora of the skin surface or respiratory tract, not classified as pathogens. The detection rate of pathogens in BALF exceeded that in tissue, with 35 cases(59.32%) showing consistent results in both sample types, 20 cases(33.90%) with pathogen detection in BALF but not in tissue, and 4 cases with exclusive detection in tissue. Among the latter, two cases revealed *Cryptococcus neoformans* in the tissue, confirmed as pulmonary cryptococcosis through tissue pathology; one case featured *Legionella pneumophila*, and one case showed *Pneumocystis jirovecii*. The initial treatment plan was adjusted based on the test results for these four patients. Species detected in both BALF and tissue, including *Streptococcus mitis*, *Haemophilus influenzae*, *Klebsiella pneumoniae*, *Prevotella melaninogenica*, and *Fusobacterium nucleatum*., exhibited higher sequence numbers in BALF than in lung biopsy tissue. For one patient, definitive diagnosis remained elusive despite CMT, pathology, and mNGS testing. The patient was eventually diagnosed with bacterial pneumonia through imaging and clinical diagnostic treatment, yet the pathogen remained unidentified. False positives also emerged in lung biopsy tissue, exemplified by a case of lung abscess with pneumonia indicating *Candida haemulonii* in the lung tissue and *Haemophilus influenzae* in BALF. The pneumonia was ultimately treated without considering the potential involvement of *Candida* in the infection. Overall, BALF demonstrated higher sensitivity but lower specificity than tissue. The most frequently detected species in both BALF and tissue were EBV, followed by *Fusobacterium nucleatum*, *Mycoplasma pneumoniae*, *Chlamydia psittaci*, *Haemophilusin fluenzae*, *Parvimonas micra*, *Porphyromona sendodontalis*, *Eubacterium brachy*, *Haemophilus parainfluenzae*, CMV.

Among patients undergoing both tissue biopsy and lavage fluid testing, 18 cases (30.51%) received a diagnosis of lung abscess with pneumonia, all confirmed through mNGS testing. Bacterial infections were present in all 18 patients, including one case of mixed bacterial and fungal infection, and one case of mixed bacterial and SARS-CoV-2 infection. Lung abscesses often result from multiple infections from dental or oral flora, typically involving weak pathogens and difficult-to-culture anaerobic or facultatively anaerobic bacteria. Notable species encompassed *Fusobacterium nucleatum*., *Prevotella spp*., *Streptococcus intermedius*, *Streptococcus anginosus*, *Porphyromonas gingivalis*, *Rothia mucilaginosa*, *Streptococcus mitis and Campylobacter showae*. Pathological findings commonly revealed focal acute suppurative changes and chronic inflammation (Table 2).

**Table 2:** Pathologic findings and final clinical diagnosis of patients with tissue and BALF mNGS were done simultaneously

Patient ID	Histopathology results	Clinical diagnosis
84	Focal chronic inflammatory changes	1.Chronic obstructive pulmonary disease with acute lower respiratory infection;2.Klebsiella pnenmoniae infection
65	Interstitial lung inflammation;organizing pneumonia	1.Pneumocystis carinii pneumonia; 2.Interstitial lung disease (post-inflammatory)
52	Focal chronic inflammatory changes	Lung abscess with pneumonia and oral candida infection
138	Focal chronic inflammatory changes	Chronic obstructive pulmonary disease with acute lower respiratory tract infection
16	Focal chronic inflammatory changes	Lung abscess with bacterial pneumonia
46	Scattered inflammatory cell infiltration in the interstitium	1. Legionella pneumophila pneumonia; 2. Type I respiratory failure; 3. Pulmonary Nocardiosis; 4. Enterococcus faecalis infection; 5. Pseudomonas aeruginosa infection; 6. Chronic Obstructive Pulmonary Disease
N424	Right middle lobe: acute inflammation of few bronchial mucosa. Right lung tissue:organizing pneumonia	1. Lung abscess with pneumonia; 2. acute exacerbation of chronic obstructive pulmonary disease; 3. streptococcal pneumonia
N500	Focal chronic inflammatory changes	1. Pneumonia with multiple infections 3. legionella pneumophila pneumonia 5. chlamydia psittaci pneumonia
N608	Focal chronic inflammatory changes	1.Chronic obstructive pulmonary disease with acute lower respiratory infection; 2. bacterial pneumonia;3. viral pneumonia;4. novel coronavirus infection (moderate);5. right pleural effusion
118	Massive lymphocyte infiltration	1. Chronic obstructive pulmonary disease with acute lower respiratory tract infection; 2. type I respiratory failure; 3. Pseudomonas aeruginosa pneumonia
N299	organizing pneumonia	Lung abscess without pneumonia
97	Interstitial lung disease with small interstitial lymphocytic infiltration, organizing pneumonia	1. Bronchiectasis with hemoptysis; 2. Haemophilus influenzae pneumonia; 3. Organizing pneumonia (secondary)
N529	Focal chronic inflammatory changes	1.Lung abscess with pneumonia; 2.hemoptysis 3.COVID-19 infection
94	Chronic inflammation, interstitial fibrous tissue proliferation and organizing pneumonia	Chronic obstructive pulmonary disease with acute lower respiratory tract infection
N388	Chronic inflammation, organizing pneumonia	1. Organizing pneumonia 2. Klebsiella pneumoniae infection 3. Pseudomonas aeruginosa infection
N317	Chronic inflammation with fibrous tissue proliferation	Lung shadows, chronic inflammatory fibrous organizing changes, bronchial asthma (acute exacerbation)
N294	organizing pneumonia	Lung abscess with pneumonia
152	Pulmonary cryptococcosis	Pulmonary cryptococcosis
12	Focal lymphocytic infiltration	Haemophilus influenzae pneumonia
43	organizing pneumonia	1.Organizing pneumonia; 2.Gram-positive bacterial pneumonia
79	Chronic inflammation of the interstitial lungs	1. Psittacosis; 2. Pseudomonas aeruginosa pneumonia; 3. Urinary tract infections
N319	Chronic inflammation of the interstitium of the lung with focal acute inflammation	1.Lung abscess with pneumonia;2.Streptococcus asteroides pneumonia
91	Focal chronic inflammatory changes	Bacterial pneumonia
N234	organizing pneumonia	Chronic obstructive pulmonary disease with acute lower respiratory tract infection,Organizing pneumonia;Mycoplasma pneumoniae
103	Chronic inflammatory changes in the interstitium of the lungs with charcoal deposition	Pulmonary cryptococcosis
N247	No obvious granulomatous changes	Mycobacterium abscessus pneumonia
N220	Acute and chronic inflammation with focal organizing pneumonia	AECOPD,Bronchiectasis with infection
N489	Lung tissue Widened alveolar septa; fibrous tissue hyperplasia; histiocyte aggregates in alveolar lumens; occasional lymphocyte distribution	1.Lung abscess with pneumonia;2.Haemophilus influenzae pneumoniae
N460	Acute and chronic inflammation with focal organizing pneumonia	1.Lung abscess with pneumonia;2.organizing pneumonia
133	Focal organizing pneumonia with partial alveolar epithelial cell hyperplasia	Lung inflammation
48	Focal chronic inflammatory changes	Bacterial pneumonia
9	Foci of mechanisation were seen in the alveolar cavities with scattered inflammatory cell infiltrates	Targeted therapy for malignant tumours
77	Chronic inflammatory with charcoal deposition in the interstitium of the lungs	1. Pulmonary non-tuberculous mycobacteriosis; 2. Mycobacterium avium infection; 3. Acute leukaemia
N502	Chronic inflammation of lung tissue	1. Sepsis;2. Klebsiella pneumonia;3. liver abscess; 4. pleural effusion
36	Squamous cell carcinoma	Malignant tumour of the left lung (squamous carcinoma)
89	Chronic inflammatory with fibrous tissue proliferation, focal organizing pneumonia	1. Klebsiella pneumoniae infection; 2. Bacterial pneumonia
120	organizing pneumonia	1.Enterococcal pneumonia; 2.Organising pneumonia; 3.Type I respiratory failure
N441	Focal organizing pneumonia with acute inflammation of small foci	Lung abscess with pneumonia
N433	organizing pneumonia	1.Organizing pneumonia;2.Mycoplasmal pneumonia
N397	Fibrinoid necrosis	1.Lung abscess with pneumonia;2.invasive adenocarcinoma of the upper lobe of the left lung pT1bN0M0 Stage IA2; 3.Klebsiella pneumoniae infection
N395	Focal chronic inflammatory changes	1. Pleurisy;2. Pleural effusion
N543	Small amount of cartilaginous tissue	1. Klebsiella pneumonia 2. Klebsiella pneumoniae infection
N522	Chronic inflammatory with deposition of carbon mill particles	Bacterial pneumonia: Streptococcus constellatus pneumonia
N334	Mucosal inflammation; Focal organizing pneumonia	Lung infections, Chlamydia psittaci, cryptococcosis
N431	Focal chronic inflammatory changes	1.Right middle lobe syndrome; 2.Lung abscess with pneumonia
N479	Chronic inflammation with acute inflammatory changes	Lung abscess with pneumonia
N218	Focal chronic inflammatory changes	Mycoplasma pneumoniae with fever
N242	Left lung: Focal chronic inflammatory changes. Right:Calcified nodules and a small amount of mucosal epithelium were seen	Haemophilus influenzae pneumonia with atelectasis
140	Chronic inflammation with acute inflammatory;Focal organizing pneumonia	1.Lung echinococcosis;2. Hepatic echinococcosis (liverworm disease); 3. Haemophilus influenzae pneumonia
N244	Focal chronic inflammatory changes	Mycoplasma pneumoniae
19	Localised lymphocytic infiltration	Streptococcus pneumoniae
145	Alveolar interstitial vasodilatation, congestion, small lymphocytic infiltration	Lung shadows: Mycobacterium kansasii infection
N372	Focal chronic inflammatory changes	1.Bacterial pneumoniae; 2.Organizing pneumonia
N457	chronic granulomatous inflammation (CGS)	1. pulmonary cryptococcosis;2. staphylococcal pneumoniae;3. pneumothorax
N614	Acute purulent changes	Lung abscess with pneumoniae
N669	Large number of foamy histiocytes, some plasma cells and lymphocytes, a small number of neutrophils infiltration	1.Streptococcal pneumoniae;2. Lung shadows
N671	Chronic Inflammation with Acute Inflammatory Mechanism Fibrous Tissue Proliferation. Focal organizing pneumonia	Lung abscess with pneumoniae
N715	Chronic inflammation of lung tissue	1.Bronchiectasis with infection;2. Fusarium Steiner lung abscesses
N717	Squamous cell carcinoma	Bronchiectasis with infection

### Detection statistics of herpesviruses

**Fig. 3:**
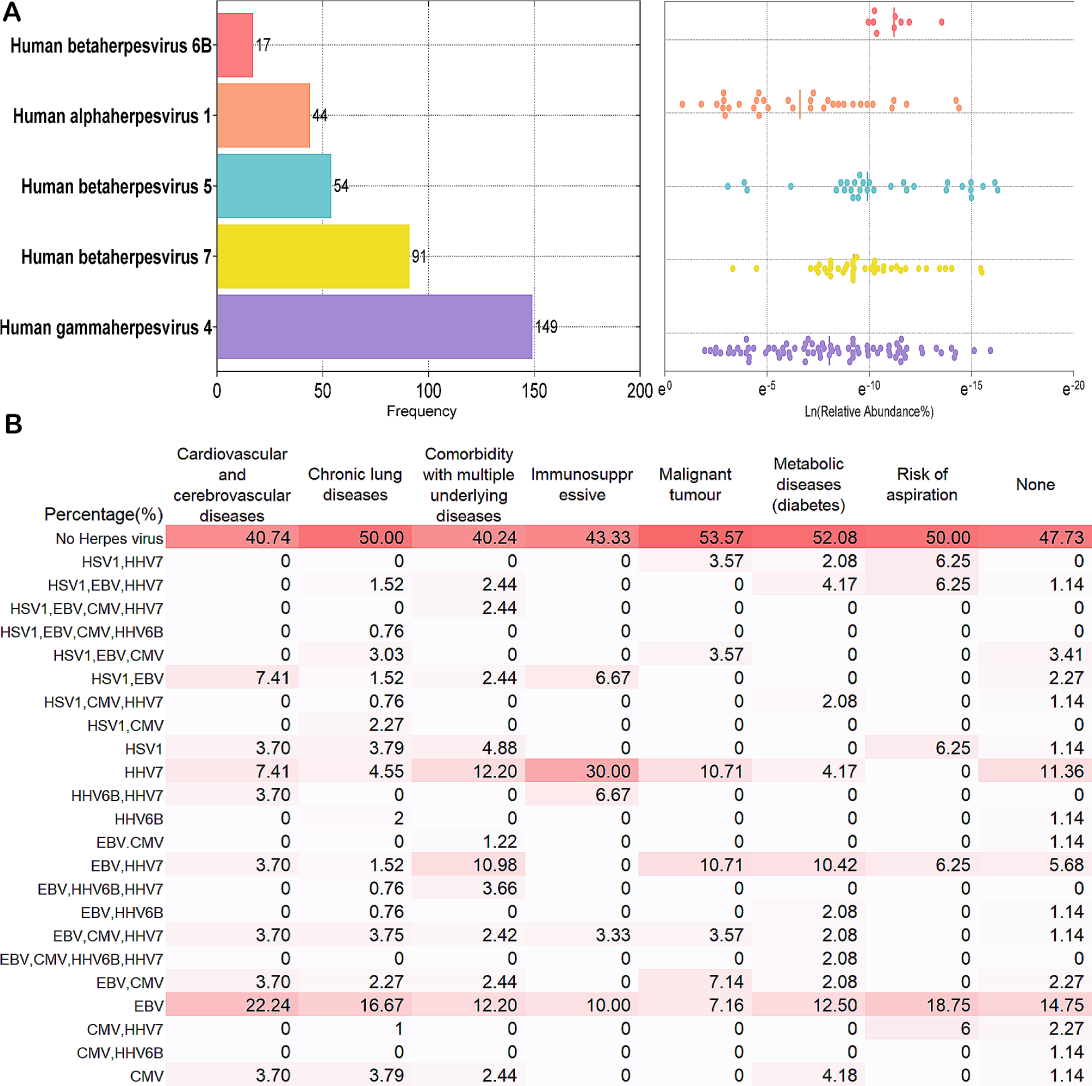
Detection Frequency and Abundance of Human Herpesviruses. (**A**) Prevalence of the five human viruses in suspected lower respiratory tract infection patients at our center, arranged by their prevalence.(Left) Viral load is presented on the x-axis as the proportion of viral sequences detected per patient sample relative to all microorganisms in that sample, with values transformed to a logarithmic scale, the bar represents the median(Right). (**B**) Heatmap illustrated the detection rates of various subtypes of herpesviruses across patient populations with different underlying diseases

Among the 451 samples subjected to mNGS, EBV was detected in 33.04% of individuals. The subsequent prevalent viruses included HHV-7 (91 occurrences, 20.18%), CMV (54 occurrences, 11.97%), HSV-1 (44 occurrences, 9.76%), and *Herpesvirus-6B* (HHV-6B) (17 occurrences, 3.77%) (Fig. 3.A). Scatter plots were created using the natural logarithm (Ln) of their relative abundances. Significant differences were observed in the relative abundances of HSV-1 compared to HHV-7 (*p* = 0.0061) and CMV (*p* = 0.0227). The median relative abundance of HHV-6B was the highest (3.03%), followed by EBV (1.02%), HSV-1 (1.02%), CMV (0.28%), and HHV-7 (0.13%) (Fig. 3B).

Subsequently, we tallied the instances of concurrent herpesvirus detection. Fluid samples from immunosuppressed hosts facilitate the easy identification of herpesviruses, leading us to conduct a statistical analysis of herpesvirus subtype variations based on the patients’ underlying diseases (Fig. 3B). EBV was the most commonly detected virus, with detection rates of 22.22% in patients with Cardiovascular and cerebrovascular diseases, 16.67% in Chronic lung diseases, 12.20% in Comorbidity with multiple underlying diseases, 10.00% in Immunosuppressed patients, 7.14% in Malignant tumor patients, 12.50% in patients with Metabolic diseases (diabetes), and 18.75% in patients at risk of aspiration. The detection rates of HHV-7 were 7.41% in patients with Cardiovascular and cerebrovascular diseases, 4.55% in Chronic lung diseases, 12.20% in Comorbidity with multiple underlying diseases, 30.00% in Immunosuppressed patients, 10.71% in Malignant tumor patients, and 4.17% in patients with Metabolic diseases (diabetes). It is notable that the proportion of immunosuppressed patients was relatively high among those with detected HHV-7. Patients with detected EBV exhibited the highest proportion of Cardiovascular and cerebrovascular diseases (33.32%) and Chronic lung diseases (25.77%). In patients with Comorbidity with multiple underlying diseases (*n* = 82), the simultaneous detection rate of EBV and HHV-7 was 10.98%, followed by Malignant tumor (*n* = 28, 10.71%) and Metabolic diseases (diabetes) (*n* = 48, 10.42%). The distribution of different herpesvirus subtypes among patients with other types of underlying diseases was relatively uniform.

### Risk factors affecting adverse prognosis in patients with LRTIs

Out of 451 LRTIs patients, 425 cases (94.24%, 425/451) demonstrated improvement, while 26 cases (5.75%, 26/451) resulted in mortality. To investigate the risk factors contributing to mortality in LRTIs patients, we initially performed a baseline differential analysis of various outcome groups (Table 3). The Mortality group exhibited a lower lymphocyte percentage than the Improvement group (6.65 Vs. 11.80, *p* = 0.003). A significant disparity in the prevalence of underlying diseases between the two groups was observed(*p* < 0.001). Although the duration from admission to mNGS testing was lengthier in the Mortality group (4 days Vs. 2 days, *P* = 0.08), the variance was not statistically significant. The distribution of CMT diagnostic results varied significantly between the two groups (*P* = 0.02). However, factors such as CRP, PCT, WBC, neutrophil percentage, CD4^+^ T cell levels, age, gender, mNGS diagnostic outcomes, infection status, mixed infection, and whether the herpes virus did not reveal significant differences between the Improvement group and the Death group (*P* > 0.05) (Table 3). Subsequently, factors with *P* < 0.10, including Age, PCT, Days from admission to mNGS testing, Lymphocyte percentage, Underlying disease, CMT, and Herpesvirus positive, underwent further evaluation through logistic regression. Initially, univariate regression analysis was conducted, revealing that an extended time from admission to mNGS testing (*P* = 0.02, OR: 1.10; 95% CI: 1.01, 1.19), low lymphocyte percentage (*P* = 0.011, OR: 0.93; 95% CI: 0.88, 0.98), the presence of underlying diseases, particularly Chronic lung diseases (*P* = 0.001, OR: 0.11; 95% CI: 0.03, 0.42), Comorbidity with multiple underlying diseases (*P* = 0.007, OR: 0.13, 95% CI: 0.03, 0.58), false negative CMT test results (*P* = 0.015, OR: 0.30, 95% CI: 0.11, 0.79), and Herpesvirus positive (*P* = 0.089, OR: 0.48, 95% CI: 0.20, 1.12) may be associated with poor prognosis in LRTIs patients (Table 4). When the *P* value in univariate analysis is less than 0.1, a multivariate regression analysis was conducted. Chronic lung diseases (*P* = 0.024, OR: 0.18, 95% CI: 0.04, 0.79) and Comorbidity with multiple underlying diseases (*P* = 0.01, OR: 0.12, 95% CI: 0.02, 0.61) were significantly associated with adverse prognosis in LRTIs patients.

**Table 3:** Characteristics of basic information in the two groups of patients enrolled in this study with improved outcomes and mortality

Variable	Total (n = 451)	Improvement (n = 425)	Mortality (n = 26)	Statistic	*P*
Age, median (Q₁, Q₃)	63.00 (46.00–75.00)	63.00 (46.00–75.00)	73.50 (55.75–79.00)	Z = 1.882	0.06
CRP, median (Q₁, Q₃)	36.05 (8.85–93.33)	34.90 (8.50–88.70)	48.70 (13.45–119.65)	Z = 1.002	0.316
PCT, median (Q₁, Q₃)	0.50 (0.22–0.81)	0.46 (0.22–0.80)	0.66 (0.41–1.99)	Z = 1.753	0.08
WBC 10^9 L, median (Q₁, Q₃)	7.76 (5.37–11.51)	7.69 (5.39–11.45)	8.56 (5.69–14.27)	Z = 1.354	0.176
Neutrophil percentage, median (Q₁, Q₃)	76.45 (63.40–86.20)	76.20 (63.60–86.20)	79.50 (63.63–87.00)	Z = 0.458	0.647
Lymphocyte percentage, median (Q₁, Q₃)	11.35 (5.60–19.50)	11.80 (6.00–19.90)	6.65 (2.70–9.65)	Z = 2.943	*0.003
CD4, median (Q₁, Q₃)	242.27 (78.78–463.30)	246.46 (79.34–464.49)	223.83 (65.57–316.17)	Z = 0.923	0.356
Days from admission to mNGS testing, median (Q₁, Q₃)	2.00 (1.00–4.00)	2.00 (1.00–4.00)	4.00 (2.00–6.00)	Z = 1.741	0.088
Sex, n (%)				χ²=0.477	0.49
Female	131 (29.05)	125 (29.41)	6 (23.08)		
Male	320 (70.95)	300 (70.59)	20 (76.92)		
Underlying disease, n (%)				-	*<.001
Cardiovascular and cerebrovascular diseases	27 (5.99)	21 (4.94)	6 (23.08)		
Chronic lung diseases	132 (29.27)	128 (30.12)	4 (15.38)		
Comorbidity with multiple underlying diseases	82 (18.18)	79 (18.59)	3 (11.54)		
Immunosuppressive	30 (6.65)	26 (6.12)	4 (15.38)		
Malignant tumour	28 (6.21)	24 (5.65)	4 (15.38)		
Metabolic diseases (diabetes)	48 (10.64)	44 (10.35)	4 (15.38)		
None	88 (19.51)	88 (20.71)	0 (0.00)		
Risk of aspiration	16 (3.55)	15 (3.53)	1 (3.85)		
Mngs, n (%)				-	0.697
False negative	73 (16.19)	70 (16.47)	3 (11.54)		
False positive	12 (2.66)	11 (2.59)	1 (3.85)		
True negative	13 (2.88)	13 (3.06)	0 (0.00)		
True positive	353 (78.27)	331 (77.88)	22 (84.62)		
CMT, n (%)				-	*0.02
False negative	219 (48.56)	213 (50.12)	6 (23.08)		
False positive	45 (9.98)	40 (9.41)	5 (19.23)		
True negative	12 (2.66)	12 (2.82)	0 (0.00)		
True positive	175 (38.8)	160 (37.65)	15 (57.69)		
Infection, n (%)				-	1
No	15 (3.33)	15 (3.53)	0 (0.00)		
Yes	436 (96.67)	410 (96.47)	26 (100.00)		
Infection Types, n (%)				-	0.221
Bacterial	314 (69.62)	299 (70.35)	15 (57.69)		
Fungal	23 (5.1)	21 (4.94)	2 (7.69)		
Mixed infection	85 (18.85)	76 (17.88)	9 (34.62)		
Non-infectious disease	16 (3.55)	16 (3.76)	0 (0.00)		
Viral	13 (2.88)	13 (3.06)	0 (0.00)		
Herpes virus positive, n (%)				χ²=2.999	0.083
No	213 (47.23)	205 (48.24)	8 (30.77)		
Yes	238 (52.77)	220 (51.76)	18 (69.23)		

Q1:The 25th percentage point

Q3:The 75th percentage point

Statistically significant values are identified in boldface

**Table 4:** Univariate and Multivariate Logistic Regression Analysis of Risk Factors for Recovery in CAP Patients

Variables	Beta	S.E	OR (95%CI)	*P*	aBeta	aS.E	aOR (95%CI)	a*P*
Age	0.01	0.01	1.01 (0.99–1.03)	0.154	0	0.01	1.00 (0.98–1.03)	0.796
PCT	0.01	0.01	1.01 (0.99–1.03)	0.323				
Days from admission to mNGS testing	0.09	0.04	1.10 (1.01–1.19)	*0.02	0	0.06	1.00 (0.89–1.11)	0.974
Lymphocyte percentage	-0.07	0.03	0.93 (0.88–0.98)	*0.011	-0.06	0.04	0.95 (0.88–1.02)	0.123
Underlying disease								
Cardiovascular and cerebrovascular diseases			1.00 (Reference)				1.00 (Reference)	
Chronic lung diseases	-2.21	0.69	0.11 (0.03–0.42)	*0.001	-1.73	0.77	0.18 (0.04–0.79)	*0.024
Malignant tumour	-0.54	0.71	0.58 (0.14–2.35)	0.449	-0.11	0.82	0.89 (0.18–4.44)	0.89
Comorbidity with multiple underlying diseases	-2.02	0.75	0.13 (0.03–0.58)	*0.007	-2.1	0.82	0.12 (0.02–0.61)	*0.01
None	-18.31	1146.38	0.00 (0.00 - Inf)	0.987	-17.87	1288.07	0.00 (0.00 - Inf)	0.989
Metabolic diseases (diabetes)	-1.15	0.7	0.32 (0.08–1.25)	0.101	-1.54	0.93	0.21 (0.03–1.31)	0.096
Immunosuppressive	-0.62	0.71	0.54 (0.13–2.16)	0.383	-1.01	0.96	0.36 (0.06–2.39)	0.292
Risk of aspiration	-1.46	1.13	0.23 (0.03–2.14)	0.199	-17.88	3424.6	0.00 (0.00 - Inf)	0.996
CMT								
True positive			1.00 (Reference)				1.00 (Reference)	
False negative	-1.2	0.49	0.30 (0.11–0.79)	*0.015	-0.74	0.59	0.48 (0.15–1.51)	0.209
True negative	-15.2	1142.05	0.00 (0.00 - Inf)	0.989	-17.48	3029.64	0.00 (0.00 - Inf)	0.995
False positive	0.29	0.55	1.33 (0.46–3.89)	0.598	0.34	0.77	1.41 (0.31–6.38)	0.659
Herpes virus positive								
Yes			1.00 (Reference)				1.00 (Reference)	
No	-0.74	0.44	0.48 (0.20–1.12)	*0.089	-0.91	0.57	0.40 (0.13–1.23)	0.11

Beta: Regression Coefficient; S.E:Standard Error; OR:odds ratio

**Fig. 4:**
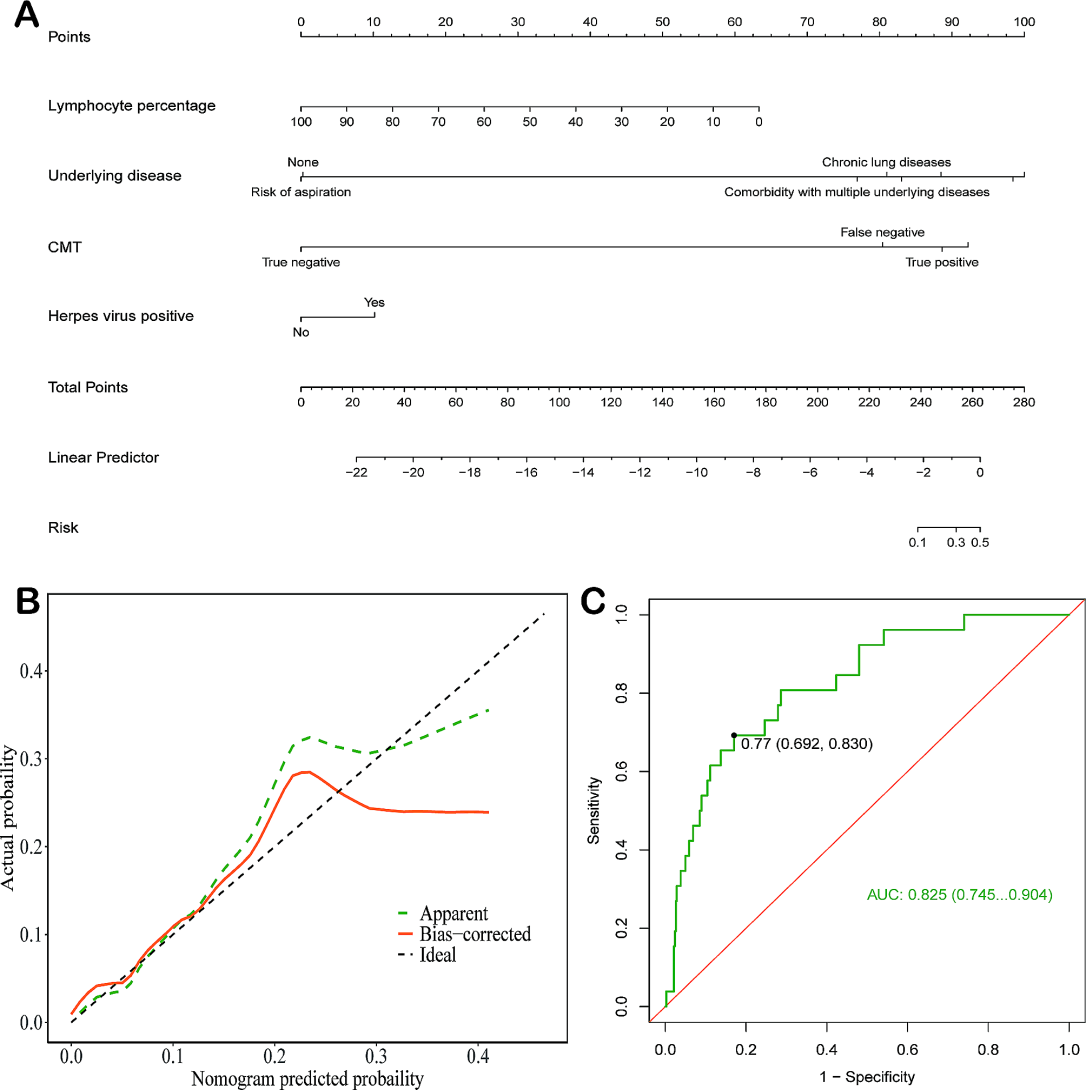
Adverse prognostic risk factors in CAP patients. (**A**) Nomogram and scoring methodology for adverse prognostic factors in CAP patients. Each predictive variable’s value (the line after each variable) corresponds to a score (top row), which is then totaled to obtain the overall score, determining the corresponding predictive probability (bottom row). (**B**) Calibration analysis of the training set, where greater alignment with the reference line indicates more precise predictions. (**C**) Nomogram model-generated ROC curve for the modeling set in predicting mortality risk in CAP patients

Subsequently, a nomogram model was developed to predict mortality risk in LRTIs patients using four significant risk factors identified through univariate regression. The left breakpoint of each scoring line segment corresponds to 0 points. A 10.00% decrease in lymphocyte proportion results in a 6.25 points increase in the nomogram model score, with a maximum value corresponding to a point of 63. The breakpoints for “Underlying disease” in “Risk of aspiration” correspond to 0 points, “None” to 0.50 points, “Chronic lung diseases” to 81 points, and “Comorbidity with multiple underlying diseases” to 77.50 points. In “CMT,” “True negative” corresponds to 0 points, “False negative” to 80 points, and “True positive” to 89 points. “Herpes virus positive” corresponds to 11 points. The total score for all variables is 402 points. As depicted in Fig. 4A, a higher total score in the nomogram model indicates an increased risk of mortality for LRTIs patients. Furthermore, the calibration curve of the modeling set (Fig. 4B) exhibits no significant deviation from the reference line, signifying strong consistency between predicted and actual values. To assess the nomogram model’s accuracy in predicting mortality risk in patients with LRTIs, a nomogram ROC curve was generated (Fig. 4C) with an AUC value of 0.825 (*P* < 0.05, 95% CI: 0.75, 0.90). This model effectively predicts the likelihood of mortality risk, with a cutoff value of 0.77 (95% CI: 0.69, 0.83).

## Discussion

LRTIs significantly impact elderly health, with 25–44 out of 1000 community-dwelling seniors contracting pneumonia annually. This risk escalates for institutionalized individuals and rises with age [25]. This retrospective study found that males represented 71% of the cases, with a median age of 63 years. The incidence of LRTIs is higher in males and escalates with age [26]. Males exhibit a greater susceptibility to LRTIs, possibly attributable to differing immune responses and behavioral factors, such as smoking [27]. Utilizing both mNGS and CMT, we identified *Klebsiella pneumoniae*, *Haemophilus influenzae*, *Streptococcus pneumoniae*, *Pseudomonas aeruginosa*, and *Staphylococcus aureus* as the predominant pathogenic microorganisms. The detection rates of the SARS-CoV-2 virus and influenza A virus were relatively high. A prospective study on Severe Community Acquired Pneumonia conducted across 17 centers in China indicated that the influenza virus was the most frequently identified pathogen in SCAP prior to the COVID-19 pandemic, with a higher prevalence during winter [28]. Bacterial infections accounted for 69.8% of cases, which is consistent with numerous studies on the etiology of CAP, despite these studies relying on culture or antibody-based methods [29]. Mixed infections were diagnosed in 18.8% of cases, highlighting an additional advantage of mNGS testing. This method is broad-spectrum, eliminating the need for prior assumptions, and enabling the detection of bacteria, fungi, viruses, and parasites in a single experiment. MNGS technology, although relatively novel, is not yet widely adopted as a primary diagnostic tool by clinicians during the early and progressive stages of disease, often being employed only after conventional methods fail. However, with growing recognition in guidelines and consensus of its high accuracy in detecting viruses, fastidious bacteria, and rare pathogens, mNGS helps reduce diagnostic delays and facilitates early targeted antimicrobial therapy. The judicious application of mNGS enhances its complementary role with CMT. Notably, the overall mNGS NPV in this study was 15.12%, surpassing CMT’s 5.19%, but significantly lower than the 50-80% reported in other studies [29–31]. These variations in results could be attributed to several factors, including differences in sample types and sampling windows, as well as the impact of antibiotic use, sample extraction library efficiency, and the consideration of sequencing costs and efficiency in pathogen sequences.Moreover, inadequate sequencing depth or filtering parameter settings could also lead to the failure in identifying low-abundance pathogens [32]. The specificity of testing tissues alone was higher than paired BALF samples (80% vs. 50%), and the NPV was also higher than BALF samples (14.81% vs. 10%).The results of concurrent testing of BALF and lung tissues revealed that false negatives detected by mNGS primarily comprised bacteria that can be easily cultured, including *Klebsiella pneumoniae*, *Escherichia coli*, *Haemophilus influenzae*, *Streptococcus pneumoniae*, Staphylococcus aureus, *Pseudomonas aeruginosa*, and *Candida albicans*.These findings aligned with previous studies [9]. On the one hand, *Escherichia coli*, *Haemophilus influenzae*, and *Streptococcus pneumoniae* are commonly colonized in the respiratory tract, and the reporting threshold of mNGS may be filtered during bioinformatic analysis. On the other hand, *Klebsiella pneumoniae*, *Pseudomonas aeruginosa*, and other easily cultured bacteria may have lower detection limits than mNGS under suitable conditions for culture. The positivity rate of lung tissue biopsies relies heavily on lesion location and sampling techniques, and is easily impacted by the host’s background. Current mNGS human depletion methods involve using saponin or other solvents for selective lysis, exposing host nucleic acids, and then utilizing a diazirine bromide compound to bind to the host nucleic acids. Subsequently, photolysis process removes the host nucleic acids and enriches the microbial reads. However, this approach is unsuitable for tissue samples [15]. Unlike liquid samples, most nucleic acids in tissues are located within the cell nucleus rather than being exposed in the cytoplasm. Furthermore, tissues contain a significant amount of connective tissue, fibronectin, collagen, and other substances, which can impact the efficacy of saponin binding. Hence, to enhance mNGS sensitivity for tissue samples, alternative methods must be explored, such as targeted sequencing, increased sequencing depth, or optimized host depletion reagents. The positivity rate of BALF exceeds that of tissue, but potential interfering factors in BALF testing, such as inadequate washing and oral bacterial contamination, could compromise test accuracy. Therefore, in cases of severe illness or inconclusive routine test results, stringent operational protocols could be adhered to for tissue collection during diagnosis. Additionally, tissue samples should be processed using a grinding method to further enhance the detection rates of fungi and tuberculosis [33].

*Haemophilus parainfluenzae*, *Porphyromonas endodontalis*, and *Fusobacterium nucleatum* were the most commonly detected bacteria in the synchronous detection of lung tissue and BALF samples. These three bacteria are components of the oral microbiota and can act as pathogens under specific conditions. MNGS technology, with its unbiased and extensive coverage in pathogen detection, frequently detects these bacteria in respiratory samples. In clinical practice, distinguishing between infection and colonization usually relies on parameters such as the number of sequences, relative abundance, and coverage of the identified microorganisms. However, the presence of oral bacteria introduced during bronchoscopy sampling often complicates the identification of pathogenic upper respiratory tract colonization in BALF. Lung biopsies not only offer deeper insights into pathological and etiological findings but also enhance the distinction between infection and colonization through histological mNGS analysis. Lung abscess can lead to necrosis of lung parenchyma [34], usually caused by the inhalation of bacteria living in the oral cavity or pharynx. The infection involves polymicrobial organisms in as high as 72.2% of cases [35], particularly including various anaerobic bacteria, but it can also be caused by a single microorganism. Collecting lung tissue specimens can provide additional insights into the pathological and etiological findings. The clinical implementation of mNGS has led to a growing number of reported cases involving lung abscesses [36] and brain abscesses [37] attributed to *Fusobacterium nucleatum*. Fusobacterium nucleatum is the predominant anaerobic bacterium among all cases of lung abscesses. *Fusobacterium nucleatum* is commonly present in the oral cavity and is generally absent or rarely detected in other parts of the body under normal circumstances [38]. This bacterium is linked to various forms of periodontal disease, as well as widespread infections and abscesses across the head, neck, brain, lungs, and abdomen [39, 40]. Previous research suggested that abscesses resulting from the synergistic activity of *Fusobacterium nucleatum* and *Streptococcus spp.* tend to be larger compared to those caused by single-bacterium infections. The primary metabolites of *Fusobacterium nucleatum* directly enhance the growth of *Streptococcus spp.*, while simultaneously impairing the ability of human polymorphonuclear leukocytes to eliminate *Streptococcus spp.* [41]. Hence, mixed infections of *Fusobacterium nucleatum* in lung abscesses might exhibit heightened invasiveness. Subsequent fundamental experimental studies can explore the interplay between bacteria and their association with the disease. *Haemophilus parainfluenzae* is a constituent of the normal flora found in the ear and oropharynx. Infrequent instances of pulmonary infection caused by this bacterium arise when *Haemophilus parainfluenzae* is inhaled from the oropharynx into the lungs, occasionally resulting in empyema [42]. Previously reported patients who suffered from pulmonary abscesses caused by *Haemophilus parainfluenzae* exhibited immunosuppression, such as diabetes [43] or malignancy [44]. In conclusion, the detection outcomes obtained through mNGS are progressively enhancing our comprehension of the pathogenic nature of commonly colonizing bacteria.

Among the 54 cases exhibiting false-negative results in routine examinations and mNGS, histopathological examination of tissue samples in some patients revealed mucosal acute or chronic inflammation, inflammatory cell infiltration, and abundant phagocytes. These patients frequently had a history of COPD, and the pulmonary infiltrates resolved gradually following comprehensive imaging and diagnostic therapeutic interventions. Potential causes of the false-negative results encompass empirical antibiotic treatment before specimen collection for testing or an exacerbation of infection resulting in elevated levels of human cells and nucleic acids in BALF, thereby influencing the sensitivity of mNGS detection. Furthermore, certain samples identified *Pseudomonas aeruginosa*, a high abundance of herpesviruses, *Pneumocystis jirovecii*, and other pathogenic microorganisms, which were subsequently determined to be false-positive mNGS results. Likely explanations for this include sample contamination by other microorganisms during specimen collection, processing, storage, and transportation; primer contamination, as primers are crucial for amplification and contamination may introduce exogenous DNA, leading to false-positive results; data analysis errors, as mNGS data analysis entails intricate bioinformatics algorithms and database comparisons, with improper parameter settings or outdated databases potentially resulting in false-positive results; sequencing errors, as despite the high accuracy of modern high-throughput sequencing technologies, occasional sequencing errors or errors caused by base substitutions may lead to misidentification of microorganisms [45]. In conclusion, when conducting mNGS, it is essential to consider various factors such as sample handling, experimental procedures, and data analysis to minimize false-positive results and enhance diagnostic accuracy.

In addition, 52.8% of the samples detected various subtypes of herpesviruses, including EBV, HHV-7, CMV, and HSV-1. Studies on the human viral group have revealed that herpesviruses are frequently detected in peripheral blood without causing symptomatic disease [46]. Herpesviruses are widely prevalent in the population and are mostly activated in response to changes in immune status. While many of the observed viruses may be common in the respiratory tract, there is insufficient research to elucidate the characteristics and pathogenicity associated with their carriage. EBV and HHV-7 were the two most commonly detected herpesviruses in this study. EBV was identified in 33.04% of the mNGS results of patients, with 50% of COPD patients (*n* = 70) carrying EBV. EBV is a significant pathogen implicated in infectious mononucleosis, lymphoma, and nasopharyngeal carcinoma, and infrequently causes pneumonia [47]. EBV prevalence is higher in patients with a history of COPD and may exacerbate viral infections in these individuals. This mechanism could involve enhanced viral survival and a diminished interferon-gamma host response [48]. Respiratory tract infections caused by EBV primarily occur in immunocompromised patients [47, 49]. Immune responses may lose control over EBV replication, leading to its reactivation and rapid replication, which is a rare but potential pathogenic factor for lung infections. HHV-6 and HHV-7 widely infect humans, with primary infections occurring in childhood. HHV-7 is present in 98% of the human population, and primary HHV-7 infection is associated with exanthem subitum, upper respiratory tract infections, and acute hepatitis in children [50]. HHV-7 detected in the lungs is recognized as a risk factor for interstitial pneumonia or idiopathic pulmonary fibrosis [51]. The positive rate of HHV-7 using PCR detection is 20.7%, and the viral load in immunosuppressed patients exceeds that in immunocompetent individuals [52]. In this research, the overall detection rate of HHV-7 is 20.18%, and 30% of immunosuppressed individuals harbor HHV-7, consistent with prior multicenter retrospective studies. 26.7% of HHV-7 positive patients exhibit clear signs of immunosuppression, but HHV-7 positivity does not impact patient prognosis [53]. Typically, herpes viruses are asymptomatic or cause mild symptoms and can be identified through blood antibodies or nucleic acids. Therefore, routine testing for respiratory pathogens or blood cultures presents challenges in determining the global prevalence of symptomatic herpes virus infections, as symptoms may be mild or not recognized as herpes virus infections [54]. Reactivation of HSV-1 in the oropharynx and subsequent shedding can lead to transmission to the lower respiratory tract through inhalation, posing a risk for LRTIs in critically ill patients [55]. CMV, HSV-1, and EBV are the most commonly reactivated viruses in the lungs of critically ill pneumonia patients. Approximately 47.4% of patients experience herpes virus reactivation, and the incidence of herpes virus viremia increases with prolonged hospitalization, with virus reactivation linked to heightened mortality risk [56]. Due to the lack of specificity in clinical criteria, radiological features, and laboratory results, definitive diagnosis of herpes virus pneumonia in respiratory samples from critically ill patients is challenging. Further research is necessary to validate the association between herpes virus and LRTIs.

In univariable risk analysis, the time from admission to mNGS testing showed a significant association with patient outcomes in lower respiratory tract infections. Each additional day before conducting mNGS testing was associated with a 10% increase in the odds of mortality (OR = 1.10, 95% CI: 1.01, 1.19, *p* = 0.02), suggesting prompt mNGS testing could correlate with improved survival by enabling targeted treatment strategies. However, this relationship was not observed in the multivariable logistic regression analysis. After adjusting for confounders such as age, sex, comorbidities, and treatment measures, the time to mNGS testing did not significantly affect the odds of mortality (β = 0, OR = 1.00, 95% CI: 0.89, 1.11, *p* = 0.974). This indicates that, when considering a range of influencing factors, the timing of mNGS testing alone does not have an independent significant impact on patient outcomes. These findings underscore the necessity to evaluate patient prognosis through a multifaceted lens, incorporating various risk factors. Further research is needed to elucidate the interplay between these factors and their collective influence on patient outcomes. A clinical prediction model for adverse prognosis in patients with LRTIs was established using logistic regression, and this model was visualized through nomograms. Despite the inclusion of 451 cases in the analysis, the proportion of death cases remained low (5.76%) due to the limitations of a single-center retrospective study. To account for potential bias, we included indicators with no significant differences (*P* > 0.1) between the death and recovery groups in the univariate logistic regression analysis. The lymphocyte percentage, which is a marker reflecting the balance of the immune system and can predict the course and prognosis of infectious diseases, is also independently associated with an increased risk of all-cause mortality [57]. Elderly COVID-19 patients are at higher risk of developing lymphocytopenia, and those with combined lymphocytopenia typically experience more severe multi-organ damage [58]. This study’s findings also reveal an association between a lower lymphocyte percentage and an increased risk of death. Furthermore, multiple studies have demonstrated that COVID-19 patients with underlying diseases, particularly diabetes and cardiovascular diseases, are at greater risk of experiencing poor outcomes [59, 60]. However, since only one patient with type 2 diabetes was among the deceased cases in this study, differences in sample size and patient baseline characteristics could lead to varying research conclusions. Additionally, the coexistence of multiple underlying diseases, particularly chronic respiratory conditions, has consistently been a leading cause of global mortality and disability, and a significant determinant of adverse patient outcomes during hospitalization [60]. Traditional methodology results are influenced by tracking patients’ conclusive diagnoses post-testing, a task often challenging to establish in the initial phases of hospitalization. Consequently, the accurate positivity of CMT relies on the clinical definitive diagnosis. Typically, CMT like culture or PCR, suggest that the pathogen load is linked to the progression or severity of the patient’s infection, with a positive result signifying a higher pathogen load. Lastly, the identification of herpes simplex virus serves as another determinant, as its reactivation signifies immunosuppression, a direct correlate to an unfavorable prognosis. Evidence of herpes simplex virus invasion of the mucosa, unexplained infiltrates, and persistent deterioration despite appropriate antibiotic treatment for ventilator-associated pneumonia, as well as histopathological changes observed in HSV-1 isolated from blood or BALF, provide clues suggesting herpes simplex virus as the causative agent. Furthermore, this viral infection is associated with an increased mortality rate among hospitalized patients [61]. Goodness-of-fit tests demonstrate a strong fit between the predicted and actual probabilities in the model. Moreover, this study generated the ROC curve of the model, yielding an AUC of 0.825. The predictive model demonstrates effective preoperative predictive ability and holds valuable clinical application. Nevertheless, this single-center retrospective study may limit the comprehensive analysis of risk factors due to uneven distribution across various groups. Future endeavors should concentrate on enlarging the sample size, augmenting the validation cohort, integrating additional clinical indicators, aiming to enhance the precision and practicality of diagnosing and prognosticating LRTIs in patients.

Despite the presence of some false positives and false negatives, mNGS can comprehensively and accurately detect pathogens at the early disease stages, offering timely support for evidence-based treatment. Particularly in the advanced phase of the COVID-19 pandemic and the escalating severity of antibiotic resistance, mixed respiratory infections are gaining increased attention. MNGS has the potential to enhance prognostic capabilities [62]. As mNGS technology becomes progressively integrated into various expert consensuses, clinicians continue to consolidate methods for the rational application of mNGS technology. It is also anticipated that technological advancements will gradually mitigate its high cost and benefit a larger population of infected patients.

## Conclusion

In summary, mNGS can rapidly obtain pathogen results, enhancing diagnostic sensitivity and efficiency. Early identification of specific pathogenic microorganisms is crucial for managing and prognosing LRTIs, with mNGS testing showing higher accuracy and superior specificity in tissue samples compared to traditional clinical microbiological testing. In cases where the pathogen cannot be clearly identified in BALF, safe collection of tissue samples for mNGS testing is recommended to ensure patient safety. However, reliance solely on mNGS results is not sufficient for definitively excluding infection. The variables in our nomogram align with prior research, indicating that a lower lymphocyte percentage, underlying diseases, and the presence of herpes virus have predictive value for adverse prognosis in patients with LRTIs.

## Data Availability

No datasets were generated or analysed during the current study.

## Abbreviations

LRTIs: Lower respiratory tract infections
BALF: Bronchoalveolar lavage fluid
CAP: Community-acquired pneumonia
CM: Galactomannan
CMT: Compared to conventional tests
*CMV*: *Cytomegalovirus*
COPD: Chronic obstructive pulmonary disease
CRP: C-reactive protein
*EBV*: *Barr virus*
*HHV-7*: *Herpes virus 7*
*HMPV*: *Human metapneumovirus*
mNGS: Metagenomic next-generation sequencing
*MTBC*: *Mycobacterium tuberculosis complex*
NPV: Negative predictive value
PCR: Polymerase chain reaction
PCT: Procalcitonin
PPV: Positive predictive value
*RSV*: *Respiratory syncytial virus*
TCR: Total consistency rate

## References

[1] Khan MN. Global, regional, and national incidence and mortality burden of non-COVID-19 lower respiratory infections and aetiologies, 1990–2021: a systematic analysis from the global burden of Disease Study 2021 GBD 2021. 2024. 10.1016/S1473-3099(24)00176-2 PMC11339187 38636536

[2] José RJ. Respiratory infections: a global burden. Annals Res Hosp 2018, 2.

[3] Kollef MH, Ward S (1998). The influence of mini-BAL cultures on patient outcomes: implications for the antibiotic management of ventilator-associated pneumonia. Chest.

[4] Kollef MH, Kollef KE (2005). Antibiotic utilization and outcomes for patients with clinically suspected ventilator-associated pneumonia and negative quantitative BAL culture results. Chest.

[5] Zhu YG, Tang XD, Lu YT, Zhang J, Qu JM (2018). Contemporary Situation of Community-acquired Pneumonia in China: a systematic review. J Transl Int Med.

[6] Chiu CY, Miller SA (2019). Clinical metagenomics. Nat Rev Genet.

[7] Wang Q, Wu B, Yang D, Yang C, Jin Z, Cao J, Feng J (2020). Optimal specimen type for accurate diagnosis of infectious peripheral pulmonary lesions by mNGS. BMC Pulm Med.

[8] Zhou Z, Li C, Zhu R, Wang D, Liu T, Jia J, Wang F, Zhao L, Dong L, Yu X, Huang H (2020). Combination of Percutaneous Lung Biopsy and Xpert MTB/RIF Ultra enhances the Differential diagnosis of tuberculosis: a prospective cohort study. Infect Dis Ther.

[9] Marr KA, Patterson T, Denning D. Aspergillosis. Pathogenesis, clinical manifestations, and therapy. Infect Dis Clin North Am 2002;16:875–894, vi. 10.1016/s0891-5520(02)00035-1 12512185

[10] Kradin RL, Mark EJ. Pathology of Pulmonary Infection Diagnostic Pathology of Infectious Disease. 2018:143–206. 10.1016/B978-0-323-44585-6.00008-4. Epub 2017 Jul 21.

[11] Shiwang H, Chong S, Hongjun L, Zhenyu Z, Liang W, Zhao G, Zhouliang W, Yuda L, Han X, Mingze T et al. Enhancing urinary tract infection diagnosis for negative culture patients with metagenomic next-generation sequencing (mNGS). Front Cell Infect Microbiol 2023, 13. 10.3389/fcimb.2023.1119020 PMC10020507 36936777

[12] Wang L, Li S, Qin J, Tang T, Hong J, Tung TH, Xu C, Yu S, Qian J (2023). Clinical diagnosis application of Metagenomic Next-Generation sequencing of plasma in suspected Sepsis. Infect Drug Resist.

[13] Zheng L, Kang Z, Wang R, Lv M, Gao Z, Xu H, Wang M (2023). Evaluation of the diagnostic performance of mNGS in detecting Intra-abdominal infections of the Emergency Department patients. Infect Drug Resist.

[14] Zhao X, Bai LP, Li BY, Yue ZZ, Zhao YC, Zhao XY (2023). Comparison of mNGS and conventional culture in non-organ transplant critically ill patients supported by ECMO: a single-center study. Front Cell Infect Microbiol.

[15] He Y, Fang K, Shi X, Yang D, Zhao L, Yu W, Zheng Y, Xu Y, Ma X, Chen L (2022). Enhanced DNA and RNA pathogen detection via metagenomic sequencing in patients with pneumonia. J Transl Med.

[16] Zhang H, Shen D, Zhou J, Yang Q, Ying Y, Li N, Cao L, Wang W, Ma X (2023). The utility of Metagenomic Next-Generation sequencing (mNGS) in the management of patients with bronchiectasis: a single-Center Retrospective Study of 93 cases. Open Forum Infect Dis.

[17] Xie F, Duan Z, Zeng W, Xie S, Xie M, Fu H, Ye Q, Xu T, Xie L (2021). Clinical metagenomics assessments improve diagnosis and outcomes in community-acquired pneumonia. BMC Infect Dis.

[18] Gu W, Miller S, Chiu CY (2019). Clinical metagenomic next-generation sequencing for Pathogen Detection. Annu Rev Pathol.

[19] Fu ZF, Zhang HC, Zhang Y, Cui P, Zhou Y, Wang HY, Lin K, Zhou X, Wu J, Wu HL (2021). Evaluations of clinical utilization of Metagenomic Next-Generation sequencing in adults with fever of unknown origin. Front Cell Infect Microbiol.

[20] Woodhead M, Blasi F, Ewig S, Garau J, Huchon G, Ieven M, Ortqvist A, Schaberg T, Torres A, van der Heijden G (2011). Guidelines for the management of adult lower respiratory tract infections–full version. Clin Microbiol Infect.

[21] Benson DA, Cavanaugh M, Clark K, Karsch-Mizrachi I, Ostell J, Pruitt KD (2018). Sayers EW: GenBank. Nucleic Acids Res.

[22] Schlaberg R, Chiu CY, Miller S, Procop GW, Weinstock G (2017). Validation of Metagenomic Next-Generation sequencing tests for Universal Pathogen Detection. Arch Pathol Lab Med.

[23] Wilson MR, Sample HA, Zorn KC, Arevalo S, Yu G, Neuhaus J, Federman S, Stryke D, Briggs B, Langelier C (2019). Clinical metagenomic sequencing for diagnosis of Meningitis and Encephalitis. N Engl J Med.

[24] Luan Y, Hu H, Liu C, Chen B, Liu X, Xu Y, Luo X, Chen J, Ye B, Huang F (2021). A proof-of-concept study of an automated solution for clinical metagenomic next-generation sequencing. J Appl Microbiol.

[25] Meyer KC. Age-associated changes in structure and function of the aging human lung. Conn’s handbook of models for human aging. Elsevier; 2018. pp. 873–88.

[26] Corica B, Tartaglia F, D’Amico T, Romiti GF, Cangemi R (2022). Sex and gender differences in community-acquired pneumonia. Intern Emerg Med.

[27] Age-sex differences (2022). In the global burden of lower respiratory infections and risk factors, 1990–2019: results from the global burden of Disease Study 2019. Lancet Infect Dis.

[28] Qu J, Zhang J, Chen Y, Huang Y, Xie Y, Zhou M, Li Y, Shi D, Xu J, Wang Q (2022). Aetiology of severe community acquired pneumonia in adults identified by combined detection methods: a multi-centre prospective study in China. Emerg Microbes Infect.

[29] Xu J, Zhou P, Liu J, Zhao L, Fu H, Han Q, Wang L, Wu W, Ou Q, Ma Y, He J (2023). Utilizing Metagenomic Next-Generation sequencing (mNGS) for Rapid Pathogen Identification and to inform clinical Decision-Making: results from a large real-world cohort. Infect Dis Ther.

[30] Chen H, Tang M, Yao L, Zhang D, Zhang Y, Zhao Y, Xia H, Chen T, Zheng J (2023). Early application of metagenomics next-generation sequencing may significantly reduce unnecessary consumption of antibiotics in patients with fever of unknown origin. BMC Infect Dis.

[31] Miao Q, Ma Y, Wang Q, Pan J, Zhang Y, Jin W, Yao Y, Su Y, Huang Y, Wang M (2018). Microbiological Diagnostic performance of Metagenomic Next-generation sequencing when Applied to Clinical Practice. Clin Infect Dis.

[32] Zhu N, Zhou D, Li S. Diagnostic Accuracy of Metagenomic Next-Generation Sequencing in Sputum-Scarce or Smear-Negative Cases with Suspected Pulmonary Tuberculosis. Biomed Res Int 2021;2021:9970817. 10.1155/2021/9970817 PMC8437628 34527747

[33] Wang C, You Z, Fu J, Chen S, Bai D, Zhao H, Song P, Jia X, Yuan X, Xu W (2022). Application of metagenomic next-generation sequencing in the diagnosis of pulmonary invasive fungal disease. Front Cell Infect Microbiol.

[34] Kuhajda I, Zarogoulidis K, Tsirgogianni K, Tsavlis D, Kioumis I, Kosmidis C, Tsakiridis K, Mpakas A, Zarogoulidis P, Zissimopoulos A (2015). Lung abscess-etiology, diagnostic and treatment options. Ann Transl Med.

[35] Moreira Jda S, Camargo Jde J, Felicetti JC, Goldenfun PR, Moreira AL, Porto Nda S (2006). Lung abscess: analysis of 252 consecutive cases diagnosed between 1968 and 2004. J Bras Pneumol.

[36] Wang N, Gao Z, He S, Han M, Han W, Liu X, Cao H, Jing M, Sun T, Xu J (2023). Lung abscess by Fusobacterium nucleatum and Streptococcus spp. co-infection by mNGS: a case series. Open Life Sci.

[37] Hu HL, Guo LY, Wu HL, Feng WY, Chen TM, Liu G (2019). Evaluation of next-generation sequencing for the pathogenic diagnosis of children brain abscesses. J Infect.

[38] Han YW (2015). Fusobacterium nucleatum: a commensal-turned pathogen. Curr Opin Microbiol.

[39] Brook I (1994). Fusobacterial infections in children. J Infect.

[40] Brennan CA, Garrett WS (2019). Fusobacterium nucleatum - Symbiont, opportunist and oncobacterium. Nat Rev Microbiol.

[41] Kuriyama T, Nakagawa K, Kawashiri S, Yamamoto E, Nakamura S, Karasawa T (2000). The virulence of mixed infection with Streptococcus constellatus and Fusobacterium nucleatum in a murine orofacial infection model. Microbes Infect.

[42] Cooney TG, Harwood BR, Meisner DJ (1981). Haemophilus parainfluenzae thoracic empyema. Arch Intern Med.

[43] Israel RH, Magnussen CR, Greenblatt DW, Patanella HP (1984). Haemophilus parainfluenzae lung abscess. Respiration.

[44] Miyamoto A, Tsuboi E, Takaya H, Sugino K, Sakamoto S, Kawabata M, Kishi K, Narui K, Homma S, Nakatani T (2006). [A case of pulmonary abscess in which Haemophilus parainfluenzae and Streptococcus intermedius were isolated by percutaneous needle aspiration]. Nihon Kokyuki Gakkai Zasshi.

[45] Wang J, Han Y, Feng J (2019). Metagenomic next-generation sequencing for mixed pulmonary infection diagnosis. BMC Pulm Med.

[46] Moustafa A, Xie C, Kirkness E, Biggs W, Wong E, Turpaz Y, Bloom K, Delwart E, Nelson KE, Venter JC, Telenti A (2017). The blood DNA virome in 8,000 humans. PLoS Pathog.

[47] Niazi MR, Iqbal QZ, Mishiyev D, Narula N, Abdul Sattar SB, Zia Z, Haider MA, Chalhoub M (2020). Epstein-Barr virus (EBV) induced pneumonitis in an immunocompetent adult: a case report. Respir Med Case Rep.

[48] McManus TE, Marley AM, Baxter N, Christie SN, Elborn JS, O’Neill HJ, Coyle PV, Kidney JC (2008). High levels of Epstein-Barr virus in COPD. Eur Respir J.

[49] Liao H, Zhu M, Cheng Z (2023). Epstein-Barr virus (EBV) induced pneumonitis in a patient with breast cancer receiving neoadjuvant chemotherapy: a case report. Respiratory Med Case Rep.

[50] Hall CB, Caserta MT, Schnabel KC, McDermott MP, Lofthus GK, Carnahan JA, Gilbert LM, Dewhurst S (2006). Characteristics and Acquisition of Human Herpesvirus (HHV)–7 infections in relation to infection with HHV-6. J Infect Dis.

[51] Yamamoto K, Yoshikawa T, Okamoto S, Yamaki K, Shimokata K, Nishiyama Y (2005). HHV-6 and 7 DNA loads in lung tissues collected from patients with interstitial pneumonia. J Med Virol.

[52] Astegiano S, Costa C, Terlizzi ME, Sidoti F, Gambarino S, Mantovani S, Solidoro P, Cavallo R, Bergallo M (2010). Detection of human herpesvirus-7 DNA in bronchoalveolar lavage. Intervirology.

[53] Xu J, Zhong L, Shao H, Wang Q, Dai M, Shen P, Xiong Y, Zhang W, Deng X, Wang M (2023). Incidence and clinical features of HHV-7 detection in lower respiratory tract in patients with severe pneumonia: a multicenter, retrospective study. Crit Care.

[54] Ayoub HH, Chemaitelly H, Abu-Raddad LJ (2019). Characterizing the transitioning epidemiology of herpes simplex virus type 1 in the USA: model-based predictions. BMC Med.

[55] Simoons-Smit AM, Kraan EM, Beishuizen A, van Strack RJ, Vandenbroucke-Grauls CM (2006). Herpes simplex virus type 1 and respiratory disease in critically-ill patients: real pathogen or innocent bystander?. Clin Microbiol Infect.

[56] Huang L, Zhang X, Pang L, Sheng P, Wang Y, Yang F, Yu H, Huang X, Zhu Y, Zhang N (2023). Viral reactivation in the lungs of patients with severe pneumonia is associated with increased mortality, a multicenter, retrospective study. J Med Virol.

[57] Fest J, Ruiter TR, Groot Koerkamp B, Rizopoulos D, Ikram MA, van Eijck CHJ, Stricker BH (2019). The neutrophil-to-lymphocyte ratio is associated with mortality in the general population: the Rotterdam Study. Eur J Epidemiol.

[58] Fei J, Fu L, Li Y, Xiang H-X, Xiang Y, Li M-D, Liu F-F, Xu D-X, Zhao H (2023). Reduction of lymphocyte count at early stage elevates severity and death risk of COVID-19 patients: a hospital-based case-cohort study. Archives Med Sci.

[59] Choi YJ, Park J-Y, Lee HS, Suh J, Song JY, Byun M-K, Cho JH, Kim HJ, Park HJ (2021). Variable effects of underlying diseases on the prognosis of patients with COVID-19. PLoS ONE.

[60] Nilav A, Karimi Rouzbahani A, Mahmoudvand G, Zavari T (2022). Evaluation of the Effect of underlying diseases on Mortality of COVID-19 patients: a study of 19,985 cases. Jundishapur J Microbiol.

[61] Pata R, Datar P (2023). The diagnosis and management of Herpes Simplex Pneumonia in the critical care setting: a Comprehensive Review. Cureus.

[62] Lu D, Abudouaini M, Kerimu M, Leng Q, Wu H, Aynazar A, Zhong Z (2023). Clinical evaluation of Metagenomic Next-Generation sequencing and identification of risk factors in patients with severe community-acquired pneumonia. Infect drug Resist.

